# Editorial: Longitudinal data analysis in child and adolescent mental health, volume II

**DOI:** 10.3389/frcha.2026.1979900

**Published:** 2026-09-14

**Authors:** Joseph M. Boden, Shuting Zheng, Jessica A. Kerr, Tomoya Hirota

**Affiliations:** 1 Department of Psychological Medicine, University of Otago Christchurch, Christchurch, New Zealand; 2 Department of Psychiatry and Behavioral Science, University of Texas at Austin, Austin, TX, United States; 3 Department of Child Development and Mental Health, Okayama University, Okayama, Japan

**Keywords:** editorial, lifecourse development, Longitudinal Studies, Mental Health, Statistical Approaches, stress

Life course theory (1) holds that development unfolds through life transitions marking periods of growth and change. Following Erikson (2), each life stage involves a developmental task whose resolution enables the transition to the next. These transitions can induce uncertainty and stress, but navigating them is critical to shaping developmental outcomes. Stress is not inherently harmful; it can stimulate growth, foster resilience, and prompt shifts in behavioural or social roles. But while some stress is normal, exposure to unpredictable or severe stressors can alter developmental trajectories in lasting ways.

This second collection on longitudinal data analysis in child and adolescent mental health focuses largely on life stress. Some of the life stress examined is tied to participants' developmental stage, some arising from mental or physical illness, and some from the Covid-19 pandemic, which disrupted daily life and social functioning worldwide.

Early childhood and the transition to school are the focus of the brief report by da Silva et al., reporting on a randomised controlled trial of Brazil's Happy Child Program, designed to promote healthy development to age six by addressing parenting behaviours. Across three assessments of 284 children and families, maternal growth monitoring and non-violent discipline were linked to positive outcomes, while older maternal age and psychological aggression predicted poorer ones.

Cai et al. used hospital record data from 94 children aged 2–5 with intellectual disabilities to track developmental quotients, a measure of progress against age norms. Cross-lagged panel modelling across three waves showed that early adaptive behaviour predicted later language and personal-social development, supporting prioritisation of adaptive skills in early intervention.

In a cohort of young Canadian children Turner et al. examine five kindergarten developmental domains: physical health, social competence, emotional maturity, language, and communication. Of 23 predictors spanning prenatal exposures to family stress, childhood family stress (at ages 1 and 3) and lower socioeconomic status predicted poorer outcomes, while prenatal maternal and postnatal child health did not.

Xu et al. focus on adolescence, a developmental period in which young people gain increasing choice and control over their diets. In a longitudinal survey of 1693 Chinese students aged 12 to 16 years, Xu et al. examined adolescent eating patterns and depressive symptoms. Skipping, or irregular, breakfast consumption and late-night snacking were significantly associated with higher levels of depressive symptoms over the course of the study. These results add to the growing evidence that diet plays an important role in adolescent development, including mental health.

Hospitalization is one potentially critical outcome for some young people experiencing mental health disorders. Aiming to improve the transition from inpatient to outpatient care, Friedrich and Zemp present a mixed-methods study protocol for the ReCoVer Study examining the role of the family environment in post-discharge outcomes following psychiatric inpatient care. Caregivers of these young people will be followed during the first six months after inpatient discharge to investigate caregiver burden and family influences on recovery.

So et al. focus on the measurement of schizotypy, a mental health disorder characterized by interpersonal deficits and related behaviour that creates difficulty for individual functioning. Using longitudinal data from over 3000 young people (15 to 24 years) in Hong Kong, their analyses confirmed a three-factor model of schizotypy, and showed that the behaviour associated with schizotypy was strongly related to psychotic-like experiences, social anxiety, and personality traits. These results highlight the potential value of schizotypy assessment for identifying and supporting young people in the early stages of serious mental illness.

Methods for diagnosing ADHD in children was the focus of the article by Himmelmeier et al, noting the challenges of obtaining consistent results across the use of multi-method examinations for diagnosis. In a sample of 125 young people aged 6 to 13 years (56 with an ADHD diagnosis), they compared self-report questionnaires, attention performance tests, and reports from parents and teachers. Parent and teacher questionnaires had the highest classification accuracy in identifying inattentive symptoms, while objective attention tests were less effective. These findings underline the importance of multi-informant assessment for the diagnosis of ADHD in young people.

Moving from mental to physical health, Zhao et al. report a longitudinal study of 60 Chinese children and adolescents (8 to 18 years) with leukemia. The study examined post-traumatic growth and coping styles over three waves of data tracking from remission to maintenance therapy. Positive coping styles were associated with greater post-traumatic growth, though gains levelled off over time. Negative coping (emotional reactivity) was associated with lower growth. The findings suggest that the assessment of coping styles and growth may support patients' wellbeing and improve outcomes among children and adolescents undergoing treatment for leukemia.

The transition to university is an important period for many young people. Han et al. followed 761 first-year students over three waves (age 17 to 19 years) to examine reciprocal associations between measures of depression and social support taken at three intervals. Simultaneous daily diary data provided additional evidence to show this effect occurring at the daily level, with depression associated with decreased support the following day, and vice versa. These results indicate that interventions addressing both factors may reduce depression and improve adjustment during the first year of university.

The Covid-19 pandemic has generated extensive research into the stress caused by disrupted education, work and family life. Xi et al. analysed data from a cohort of parents and young people on household chaos and youth emotional wellbeing at three points during the pandemic. Higher levels of perceived household chaos were associated with increasing behavioural problems, sleep disturbances, anxiety and emotional dysregulation. Older children's perceptions of household chaos tended to differ from their parent's view (unlike younger children). This research suggests that family dynamics play an important role in buffering unusual stressors and may represent an important target for interventions.

Mousavi et al. examine how adverse childhood experiences (ACEs) shaped young adults' (mean age 22 years) mental health across pre-, peak-, and post-peak pandemic periods. Among 171 participants, higher ACE exposure predicted poorer mental health under high pandemic stress and slower improvement over time — reinforcing that ACEs heighten vulnerability to later mental health problems under significant stress.

Finally, Greenwood et al. examine parental substance use during Australia's exceptionally prolonged pandemic restrictions. Among 560 parents, prior substance use predicted continued use during the pandemic, and poorer parent-infant bonding was linked to a greater likelihood of alcohol, tobacco and drug use, highlighting family systems as a target for reducing substance use during major stressors.

Together, these twelve articles showcase the depth of contemporary life course research, spanning diverse questions, methods and analytic approaches to stress and adaptation. They demonstrate both the close interrelation of life transitions and stress, and the capacity of longitudinal studies to disentangle these dynamics — informing interventions that promote resilience and adaptive functioning across the lifespan.
